# VEXAS syndrome: is it more a matter of inflammation or hematopoietic clonality? A case series approach to diagnosis, therapeutic strategies and transplant management

**DOI:** 10.1007/s00277-025-06207-2

**Published:** 2025-01-17

**Authors:** Alessandro Costa, Federica Pilo, Martina Pettinau, Matteo Piga, Pietro Carboni, Eugenia Piras, Clara Targhetta, Rodrigo Rojas, Paola Deias, Olga Mulas, Giovanni Caocci

**Affiliations:** 1 Hematology, Department of Medical Sciences and Public Health, University of Cagliari, Cagliari, 09121 Italy; 2 Hematology and BMT Unit, “A. Businco” Hospital, ARNAS Brotzu, Cagliari, Italy; 3 Rheumatology, Department of Medical Sciences and Public Health, University of Cagliari, Cagliari, 09121 Italy; 4 UOC Reumatologia, Azienda Ospedaliero Universitaria Cagliari, Monserrato, CA ss 554, 09032 Italy

**Keywords:** VEXAS syndrome, UBA1, Exon 14, Inflammation, Hematopoietic cell transplant

## Abstract

VEXAS syndrome is a complex hemato-inflammatory disorder, driven by somatic mutations in the *UBA1* gene within hematopoietic precursor cells. It is characterized by systemic inflammation, rheumatological manifestations, and frequent association with myelodysplastic syndrome (MDS). We present a series of four VEXAS cases, all of which include concomitant MDS, each displaying distinct genetic signatures and clinical features at diagnosis, with a focus on their diagnostic and therapeutic implications. Our findings underscore the importance of extending *UBA1* sequencing beyond exon 3 in cases with strong clinical suspicion. Given the rarity of non-canonical variants and the limited gene annotation, germline tissue control should be considered to differentiate somatic from germline mutations. Hematological management, including considerations for transplantation, was primarily guided by the Revised International Prognostic Scoring System (IPSS-R) for MDS due to the absence of a specific risk stratification system for VEXAS or therapy guidelines. A critical point of our discussion is the role of inflammation in the peri-transplant period; in one patient, the combination of disease-modifying antirheumatic drugs (DMARDs) and high-dose corticosteroids before transplant was crucial in controlling inflammation, resulting in a successful hematopoietic stem cell transplantation (HSCT). In contrast, uncontrolled inflammation contributed to the peri-transplant death of another patient. These cases highlight the importance of effective inflammation management in optimizing HSCT outcomes. Additionally, our study emphasizes the urgent need for specific management guidelines for VEXAS syndrome, including a comprehensive risk stratification system and optimal timing for transplantation.

## Introduction

VEXAS syndrome (Vacuoles, E1 Enzyme, X-linked, Autoinflammatory, Somatic), a rare clonal hemato-inflammatory disorder, has recently garnered attention due to its combination of inflammatory rheumatologic manifestations and hematologic abnormalities, including myelodysplastic syndromes (MDS) and plasma cell dyscrasias [1]. Diagnosis relies on detecting somatic mutations in the X-linked *UBA1* gene in hematopoietic precursor cells, encoding for the ubiquitin-like modifier activating enzyme 1 and involved in the ubiquitination process [1–2]. Mutations at Methionine-41 (p.Met41) in exon 3, such as p.Met41Thr, p.Met41Val, and p.Met41Leu, account for over 80% of cases [3]. Less common mutations include c.118-1G > C in the exon 3 splice site and c.167 C > T (p.Ser56Phe) [4]. Recently, mutations in non-exon 3 sites, such as exon 14 and exon 4, have also been reported [5–7]. Sanger sequencing remains a reliable method for identifying common variants, especially at codon 41. In contrast, next-generation sequencing (NGS) offers the advantage of detecting a wider range of mutations, including rarer non-canonical variants, though limited by higher costs and turnaround times [5, 6].

*UBA1* mutations have significant pathogenetic implications due to the associated dysregulation of the inflammatory response. Aberrant signaling of chemokine receptor and cytokine, particularly IL-1β, along with elevated TNF-α and abnormalities in circulating immune cells, drive chronic inflammation and activate programmed cell death pathways [8, 9]. This pro-inflammatory cell death may contribute to resistance to conventional anti-inflammatory rheumatologic therapies. Current treatment strategies for inflammatory symptoms include corticosteroids, disease-modifying antirheumatic drugs (DMARDs), anti-IL-1 and IL-6 biologics, and JAK inhibitors. Management of concomitant MDS, with transfusion support, erythropoiesis-stimulating agents (ESAs), and hypomethylating agents (HMAs), currently relies on approaches adapted from standard protocols for non-VEXAS-associated MDS, based on the Revised International Prognostic Scoring System (IPSS-R) [10]. Allogeneic hematopoietic stem cell transplantation (HSCT) offers potential curative benefits, especially in refractory cases, though careful patient selection and rigorous risk-benefit analysis are crucial due to potential transplant-related morbidity and mortality [11]. In this report, we share our experience gathered in managing four patients with VEXAS syndrome, focusing on two critical aspects: clonal hematopoiesis and inflammation, emphasizing the importance of addressing the latter to improve clinical outcomes, with particular attention to an effective pre-transplant regimen and optimal timing for transplantation.

## Materials and methods

### Study population

The study included four patients diagnosed with VEXAS syndrome at our institution from February 2023 to September 2024. The inclusion criteria were: (1) a confirmed diagnosis of VEXAS syndrome based on clinical presentation and *UBA1* gene mutation; (2) availability of comprehensive clinical and genetic data; (3) follow-up data on management and treatment outcomes. Four patients met these criteria and were included (Table 1). One patient was excluded due to carrying only the p.Met41Thr variant without morphological abnormalities and clonality indicative of MDS or plasma cell disorder.

**Table 1:** Demographic and clinical characteristics of enrolled patients

	Patients			
	1	2	3	4
**Clinical characteristics**				
Age of onset, years	60	54	71	68
Comorbidities	T2D, HBP	History of substance abuse; prior HCV infection	T2D, retinal detachment, HBP; dyslipidemia	Asthmatic bronchitis
Arthritis	+	-	-	-
Fever	+	+	+	-
ENT lesions	-	Periorbital and palpebral edema	-	-
Lung infiltrates	-	Lung nodule	-	-
Skin lesions	Sweet’s syndrome	Cutaneous nodules and migratory polyarthritis	Sweet’s syndrome	Sweet’s syndrome
Periorbital edema	+	-	-	-
DVT	-	-	-	Recurrent DVT
**Hematological features**				
Hematological diagnosis	MDS-IB2	MDS-LB	MDS-LB	MDS-LB
IPSS-R risk score	Intermediate-2	Intermediate-1	Low	Low
IPSS-M risk score	NA	Moderate-high	Low-moderate	NA
Bone marrow vacuoles	Myeloid and erythroid	Myeloid and erythroid	Myeloid and erythroid	Erythroid
Marrow blasts, %	10–15	3	3–4	6–7
Cytogenetic analysis	46, XY [20]	46, XY [20]	46, XY [20]	46, XY [20]
**Laboratory findings**				
Hemoglobin, g/dL	12.0	7.1	10.4	8.4
MCV, fL	82.0	96.5	90	91
WBC, x10^9^/L	18.8	3.0	3.2	1.6
ANC, x10^9^/L	14.0	0.9	2.3	0.7
Platelet count, x10^9^/L	172.0	78.0	75.0	60
EPO levels, mU/mL	35.8	-	109.2	28.7
**Genetic features**				
*UBA1* mutation	c.1430G > C, p.Gly477Ala	c.167 C > T, p.Ser56Phe	c.122 T > C, p.Met41Thr	c.122 T > C, p.Met41Thr
Co-mutation detected, VAF%	-	*SRSF2*, 30%	*ASXL1*, 35%	-
**Treatment administered**				
**Inflammation control treatments**				
First line	Prednisone + Tocilizumab	Prednisone	Prednisone	Prednisone
Second line	Prednisone + Upadacitinib	Prednisone		
Third line	Prednisone + Anakinra			
**Hematologic treatment for MDS**				
ESA	-	-	Epoetin zeta	Epoetin zeta
Transfusion	-	+	+	+
Allogeneic HSCT	+	-	-	-

Abbreviations: ANC, absolute neutrophil count; ESA, erythropoiesis-stimulating agent; GCs, glucocorticoids; HBP, high blood pressure; HCV, hepatitis C virus; HSCT, hematopoietic stem cell transplant; IPSS-M, molecular international prognostic score system; IPSS-R, revised IPSS; MCV, mean corpuscular volume; MDS-IB2, myelodysplastic syndrome with increased blasts 2; MDS-LB, myelodysplastic syndrome with low blast; MDS/MPN-N, myelodysplastic syndrome/myeloproliferative neoplasia with neutrophilia; T2D, type 2 diabetes mellitus; VAF, variant allele frequency

Diagnoses of associated myeloid disorders in VEXAS followed the 5th edition World Health Organization (WHO) classification [12]. The risk assessment for disease progression was conducted using the revised International Prognostic Scoring System (IPSS-R) for MDS [13], or when molecular data were available, the molecular IPSS (IPSS-M) [14].

Clinical data were extracted from medical records and included: patient demographics, clinical symptoms, laboratory results, bone marrow morphology, cytogenetic analysis, and flow cytometry results. Emphasis was placed on documenting systemic inflammatory symptoms, hematological anomalies, and therapy response. Patient medical histories were reviewed to assess the timing and progression of symptoms and any treatment-related complications. Adverse events were evaluated according to the Common Terminology Criteria for Adverse Events (CTCAE 5.0). All procedures in this study were conducted in accordance with the ethical principles of the Declaration of Helsinki.

### Genetic analysis

Mutations in the *UBA1* gene were identified through analyses conducted on DNA samples obtained from both peripheral blood and bone marrow. For the screening of *UBA1* mutations, various techniques were employed depending on laboratory availability, including Sanger sequencing and Next Generation Sequencing (NGS). Sanger sequencing was performed using specific oligonucleotides (Eurofins Genomics). DNA was amplified in a reaction mixture with UBA1_F and UBA1_R primers, PCR buffer, MgCl2, dNTPs, and Taq polymerase (Applied Biosystems). PCR products were purified using the Bigdye Terminator Cycle Sequencing Kit and analyzed with SeqStudio Genetic Analyzer. NGS was performed using the KAPA HyperCap Workflow v3.0 ROCHE capture method and the NextSeq550 Illumina platform. Read alignment (BWA/DRAGEN Germline), variant calling (GATK v4.2), annotation (ANNOVAR), and prioritization of variants relative to the GRCh38 reference genome were performed using the GenomeUp platform, adhering to the guidelines of the American College of Medical Genetics and Genomics (ACMG) [15]. Concomitant mutations in myeloid genes were also assessed using NGS on the Ion Torrent GeneStudio S5 platform, allowing for a comprehensive analysis of the mutational profile associated with VEXAS syndrome analyzing the whole *UBA1* gene.

## Results

### Case 1

**Fig. 1:**
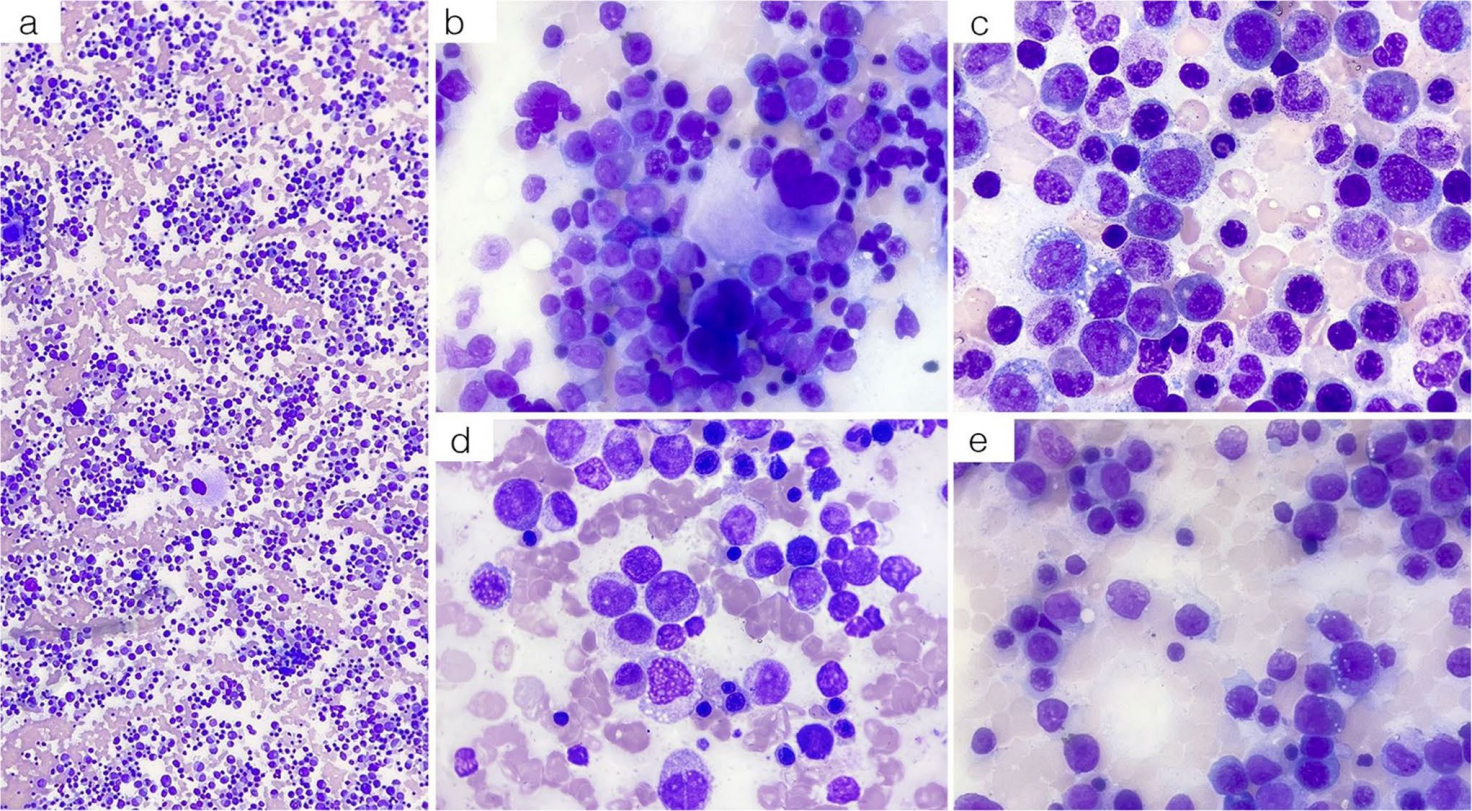
Bone marrow smear of Patient 1. The smear, examined at 10x magnification, showed hypercellularity (**a**). At 40x magnification, multilineage dysplasia was evident with a reduced erythroid series (**b**), mainly comprising dysplastic maturing erythroblasts. The granulocytic series was hyperplastic (**b**), with a left-shifted maturation curve. Cytoplasmic vacuoles were observed in erythroid and myeloid precursors (**c**, **d**, **e**). The blast count was estimated at 10–15%. Megakaryocytes exhibited significant dysplasia (**d**), with hypolobated and irregular nuclei (**b**)

A 60-year-old male presented with asthenia, erythematous skin lesions, arthritis, periorbital edema, fever, and weight loss, partially responsive to 25 mg daily prednisone. Upon admission, laboratory findings showed a hemoglobin (Hb) level of 12 g/dL, a mean corpuscular volume (MCV) of 82 fL, neutrophilic leukocytosis with an absolute neutrophil count (ANC) of 14.0 × 10⁹/L, and a normal platelet count while on glucocorticoid therapy. Inflammatory markers were markedly elevated, with increased ferritin and α-2 globulins, while the autoimmune panel was negative. Bone marrow cytomorphology revealed trilineage dysplasia, hyperplasia of the myeloid lineage, and a left-shifted maturation curve (Fig. 1). Cytoplasmic vacuoles were present in erythroid and myeloid precursors, and Perls’ staining was negative for ring sideroblasts. Blast cells were < 5%. Cytogenetic analysis showed a normal male karyotype, and flow cytometry yielded no diagnostic anomalies. Initial histopathological findings were consistent with myelodysplastic/myeloproliferative syndrome with neutrophilia (MDS/MPN-N). However, a subsequent pathological review of the same sample raised concerns about the original diagnosis, as the findings could not be definitively attributed to the primary marrow-derived clonal disorder, leading to a revised diagnosis of MDS.

Further rheumatological evaluation of the skin lesions, supported by histopathological analysis, led to the diagnosis of histiocytoid Sweet’s syndrome. Given the combination of both rheumatologic and hematologic abnormalities, alongside partial steroid resistance, *UBA1* gene mutation analysis was performed. Sanger sequencing for canonical mutations in exon 3 was negative; however, NGS revealed a non-canonical mutation, c.1430G > C; p.Gly477Ala, in exon 14, confirming the diagnosis of VEXAS.

To manage symptoms, low-dose prednisone (25 mg once daily) was administered in combination with weekly subcutaneous tocilizumab (162 mg), following the recommendation of the rheumatology team. Despite some initial improvements, arthritis, skin lesions, and anemia persisted, prompting the initiation of a polytransfusion regimen. After four months, tocilizumab was discontinued due to grade 2 neutropenia and an incomplete clinical response. Treatment with the JAK inhibitor upadacitinib (15 mg once daily) was then started, allowing for steroid tapering and further symptom improvement. This treatment course was complicated by pancytopenia which required hospitalization, and the re-emergence of rheumatologic symptoms following treatment withdrawal. Anakinra (100 mg once daily) was added alongside steroids but was gradually tapered and eventually discontinued due to grade 3 neutropenia.

Due to the worsening hematologic parameters and persistent inflammatory symptoms, the patient was re-evaluated through bone marrow biopsy, which revealed a blast percentage of 10–15%. Considering the observed disease progression and the availability of an HLA-identical female family donor, the patient was deemed eligible for allogeneic HSCT, and peripheral blood selected as the source of hematopoietic stem cells. After completing the evaluation for transplantation and in agreement with the rheumatology team, the patient received two high-dose steroid boluses (methylprednisolone 3 mg/kg), with the first bolus combined with intravenous tocilizumab (600 mg) as peri-transplant management. This treatment resulted in symptom remission and a reduction in inflammatory markers. The conditioning regimen included treosulfan, fludarabine, and thiotepa, with graft-versus-host disease (GvHD) prophylaxis based on anti-thymocyte globulin, methotrexate, and cyclosporine. During hospitalization, the patient developed grade 3 oropharyngeal and gastrointestinal mucositis, febrile neutropenia associated with pulmonary inflammation, and a *Pseudomonas aeruginosa* infection confirmed by blood culture. Despite these complications, the transplantation was successful, with complete engraftment and full donor chimerism observed by day + 20 post-transplant, and no signs or symptoms of GvHD. Post-transplant *UBA1* reassessment through NGS has been scheduled.

### Case 2

A 54-year-old male with a history of substance use disorder and prior hepatitis C virus (HCV) infection, initially treated with interferon and later with glecaprevir/pibrentasvir, presented at our Institution for severe pancytopenia (Table 1), prompting further diagnostic investigation.

Bone marrow smear revealed trilineage dysplasia with low blast count (< 5%) and rare vacuoles in both myeloid and erythroid progenitors. The bone marrow biopsy showed erythroid hyperplasia with atypical paratrabecular localization, and a left-shifted, hyperplastic myeloid lineage predominantly distributed in the interstitial space. Reticulin staining demonstrated a diffuse increase in fibrosis (MF-2). Mutational analysis for *JAK2*, *CALR*, and *MPL* tested negative. Cytogenetic analysis revealed a normal karyotype, while NGS identified an *SRSF2* mutation (c.284_307del; p.Pro95_Arg102del) with a variant allele frequency (VAF) of 43%. The diagnosis of MDS with low blasts (MDS-LB) was then established.

A rheumatology consultation was requested due to clinical signs suggestive of VEXAS syndrome, including migratory arthritis, skin nodules, edema, and eyelid swelling, which partially responded to intermediate-dose steroids. Subsequent *UBA1* gene analysis confirmed the somatic S56F variant. The low blast count precluded initiation of treatment with HMAs, limiting management to red blood cell (RBC) transfusion support. However, due to persistent symptomatic burden and ongoing RBC-transfusion-dependence, although classified as intermediate-1 risk according to the IPSS-R, the patient was considered for transplantation. A search for a matched unrelated donor was initiated, as no familial donor was available.

**Fig. 2:**
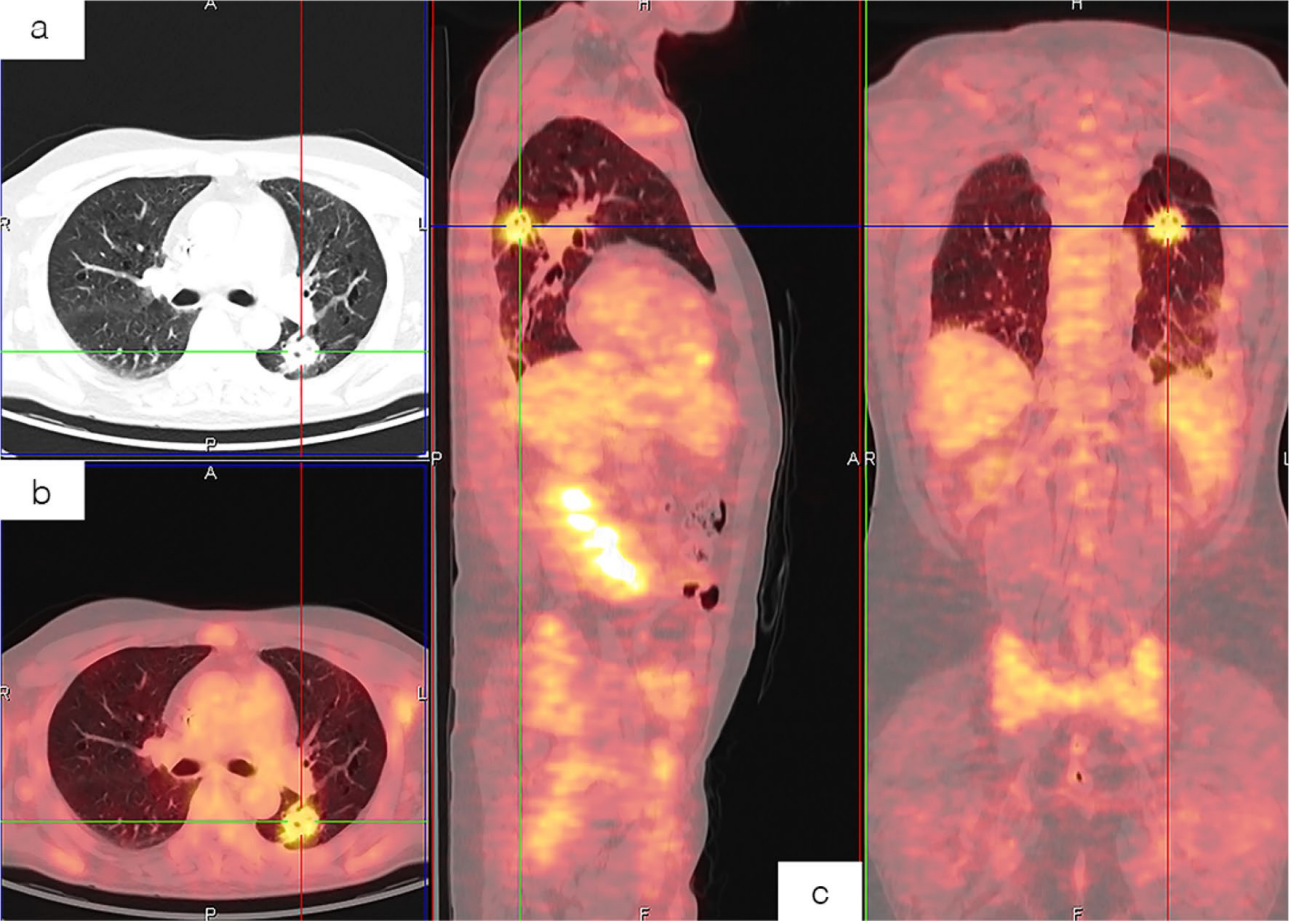
^18^FDG PET/CT imaging for the characterization of a pulmonary nodule in VEXAS syndrome. The examination demonstrated heterogeneous radiotracer uptake, characterized by a central area of hypometabolism within the irregular lesion located in the apical segment of the left lower lobe. Concurrently, diffuse hypermetabolic activity was observed in the bone marrow, accompanied by widespread and heterogeneous hepatosplenic uptake, without any distinct focal lesions identified

Symptoms management included low-dose prednisone (25 mg once daily) while supplementation with anakinra was considered, but not established due to a high risk of infection. However, inflammatory symptoms and markers remained uncontrolled. During pre-transplant hospitalization, the patient’s condition worsened, marked by fever and an ulcerative skin lesion positive for *Pasteurella* spp., progressing to bacteremia with pulmonary involvement. An ^18^F-FDG PET-CT scan was performed to further evaluate a previously identified pulmonary nodule. The scan revealed extensive metabolically active disease, with involvement of both supradiaphragmatic and infradiaphragmatic lymph nodes, as well as bone marrow infiltration (Fig. 2). A biopsy of the pulmonary nodule, performed due to concern for a neoplastic lesion, showed macrophages, sparse lymphocytes, alveolar remnants, and extensive necrosis, consistent with the pulmonary manifestations of VEXAS as previously described [1, 16]. Meanwhile, the pulmonary condition continued to worsen, with the appearance of numerous sub-centimetric nodules. Additionally, despite severe thrombocytopenia, thrombotic formation was documented in the right ventricle, deemed inoperable, and complicated by the onset of left atrial fibrillation. These events contributed to the patient’s clinical deterioration, ultimately leading to a fatal outcome.

### Case 3

A 71-year-old male with a medical history significant for Sweet syndrome, type 2 diabetes mellitus (T2D), hypertension, and hypercholesterolemia initially presented to our institution for evaluation of a monoclonal component detected during routine screening. Subsequent investigations confirmed a diagnosis of IgMκ MGUS. Three years later, the patient was re-evaluated due to worsening non-deficiency macrocytic anemia, with a Hb level of 8.8 g/dL and MCV of 104 fL. Bone marrow examination revealed erythroblastic hyperplasia with left-shifted maturation and an increased number of dysplastic megakaryocytes. Cytoplasmic vacuolization was noted in both erythroid and myeloid precursors. Histopathological analysis showed a moderate increase in reticulin fibers. Cytogenetic studies confirmed a normal male karyotype, while NGS identified a pathogenic variant in the *ASXL1* gene (c.C2668T; p. L890F; VAF 35%).

Consequently, the patient was diagnosed with MDS-LB and initiated treatment with ESA and deferasirox to manage iron overload. Despite these interventions, his anemia continued to worsen, resulting in increased transfusion dependency and markedly elevated serum ferritin levels (1423 ng/ml). Given the history of Sweet syndrome and concomitant inflammatory symptoms, genetic testing for *UBA1* mutations was performed, revealing the *UBA1* M41T. The patient was also treated with low-dose steroids (prednisone 15 mg once daily), carefully chosen given his age and comorbid conditions; however, this approach failed to provide adequate symptom control. Unfortunately, the patient’s clinical condition progressively worsened, and he ultimately passed away due to further deterioration and exacerbating heart failure during rehabilitation at a long-term care facility.

### Case 4

A 68-year-old male with a history of tuberculous pleuritis and deep vein thrombosis (DVT) complicated by pulmonary embolism presented with progressive dyspnea, asthenia, and severe anemia. Initial hematological evaluation revealed marked pancytopenia, along with elevated erythrocyte sedimentation rate and C-reactive protein levels. Bone marrow examination demonstrated dysplastic erythroid hyperplasia with a myeloid blast count of 4%, leading to a diagnosis of MDS-LB. No vacuoles were identified in hematopoietic precursors at that time.

During follow-up, the patient experienced recurrent DVT, for which the direct oral anticoagulant (DOAC) rivaroxaban was initiate. Thrombophilia screening, including tests for protein C, protein S, antithrombin III levels, factor V Leiden mutation, prothrombin G20210A mutation, and antiphospholipid antibodies, was negative. In accordance with the IPSS-R risk stratification, the patient’s RBC-transfusion-dependence and low erythropoietin levels, ESA therapy was initiated. Additionally, low-dose prednisone (25 mg once daily) was administered for a chronic dermatitis characterized by erythematous, infiltrative lesions suggestive of Sweet’s syndrome.

Given the atypical clinical features and the confirmed diagnosis of Sweet’s syndrome, a repeat bone marrow evaluation was performed. This reassessment revealed the presence of cytoplasmic vacuoles solely in erythroid precursors, with no evidence of involvement in myeloid lineage cells. Genetic testing for *UBA1* mutations confirmed the Met41Thr mutation in exon 3, establishing a diagnosis of VEXAS syndrome. However, the patient’s clinical course was further complicated by acute heart failure, necessitating transcatheter aortic valve implantation. Subsequently, he developed infectious complications secondary to ischemic-necrotic lesions affecting the first four toes of the right foot due to chronic obliterative arteriopathy, requiring transfemoral amputation of the right lower limb. Postoperative recovery was complicated by the onset of macrophage activation syndrome, ultimately leading to the patient’s death.

## Discussion

Historically, the management of MDS have focused on underlying clonality, bone marrow failure, and the risk of progression to acute myeloid leukemia. However, VEXAS syndrome challenges this paradigm. The cases presented highlight how managing these patients requires careful consideration of the role of hematopoietic clonality and the clinical consequences of the systemic inflammatory process.

Despite increasing awareness of clinical “red flags,” diagnosis can be challenging due to confounding factors related to the underlying inflammation. For instance, in patient 1, inflammation and concurrent steroid therapy likely masked the clinical picture, initially leading to a misinterpretation as MDS/MPN-N. Indeed, neutrophilia is not uncommon in VEXAS, and dysplastic leukocytes have been observed in the peripheral blood of these patients [17]. This underscores the need to critically distinguish the effects of the inflammatory process and/or ongoing therapy, even in the presence of an underlying clonal disorder.

**Fig. 3:**
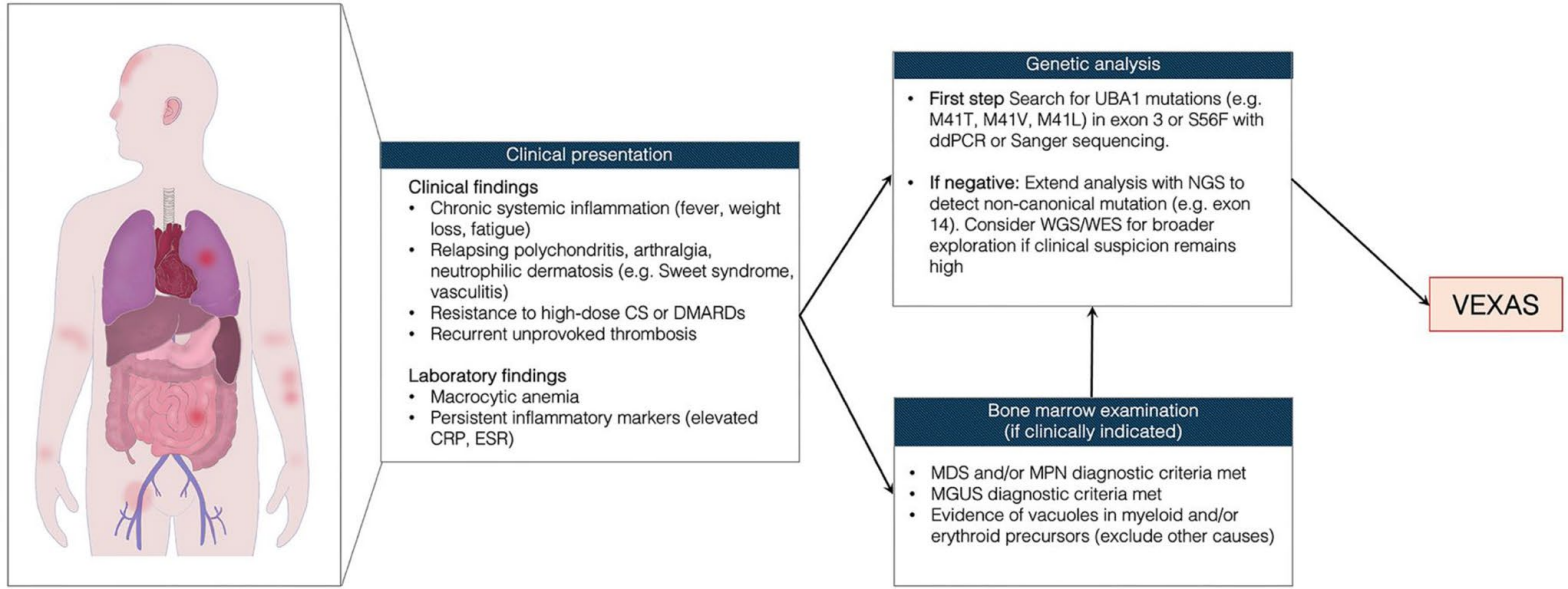
Diagnostic flow-chart for VEXAS syndrome. Abbreviations: CRP, C-Reactive Protein; CS, corticosteroids; ddPCR, digital droplet PCR; DMARDs, disease-modifying antirheumatic drugs; ESR, erythrocyte sedimentation rate; MDS, myelodysplastic syndrome; MPN, myeloproliferative neoplasms; MGUS, monoclonal gammopathy of unterminated significance; NGS, next-generation sequencing; WES, whole exome sequencing; WGS, whole genome sequencing

The same patient also carried the recently identified *UBA1* p.Gly477Ala mutation in exon 14 [5, 18]. Though rare, these *UBA1* non-canonical variants suggest a genetic complexity that may be overlooked by first-line methods like Sanger sequencing. For patients with strong clinical suspicion of VEXAS but no mutations in exon 3, advanced techniques such as NGS should be considered (Fig. 3), and germline tissue control should be performed to differentiate somatic from germline mutations [5]. These approaches enable a more comprehensive evaluation of the *UBA1* gene, potentially identifying both known and novel pathogenic variants in previously unexplored regions.

Two of our patients also tested positive for *ASXL1* and *SRSF2* mutations through NGS. Co-mutations in well-known leukemia driver genes, such as *DNMT3A* and *TET2*, are frequently reported, along with mutations in *ASXL1*, *NRAS*, *SF3B1*, and *SRSF2*. In this context, *UBA1* could represent the primary clone, a subclonal event in combination with known leukemia driver mutations, or a secondary main driver event following treatment [3, 6, 19]. Data have shown a correlation between mutations in spliceosome genes (e.g., *SF3B1*, *SRSF2*, *U2AF1*, etc.) and activation of the NF-κB pathway, along with increased pro-inflammatory cytokines [20]. These mutations may therefore contribute to the profound immune dysregulation observed in VEXAS. However, given the distinct pathophysiological mechanisms of VEXAS compared to other forms of MDS, further studies are needed to clarify the role of concomitant clonal mutations in the pathogenesis and progression of this syndrome.

Closely linked to the inflammatory process, thrombotic events are a significant concern, reported in 49% of 119 patients in a Mayo Clinic cohort [21]. While systematic screening for *UBA1* mutations in men over 50 with unprovoked thromboses is not yet recommended, the diagnosis should be considered when systemic inflammation and hematological abnormalities are present [22]. Notably, recurrent DVTs were observed in 41% of cases and in 20% during anticoagulant therapy in the Mayo Clinic study [21]. In our series, patient 4 experienced recurrent DVT episodes both prior to and following diagnosis of VEXAS, despite treatment with DOACs and corticosteroids. Additionally, despite profound thrombocytopenia, patient 2 developed an intra-atrial thrombus, which delayed HSCT and ultimately led to fatality. This suggests that the potent pro-inflammatory mechanisms in VEXAS might circumvent the protective effects of anticoagulants by activating pathways beyond direct inhibition of factors II and Xa [21, 23]. Our findings and existing literature underscore the urgent need for further studies to assess the efficacy of targeted anticoagulant therapies in VEXAS patients.

**Fig. 4:**
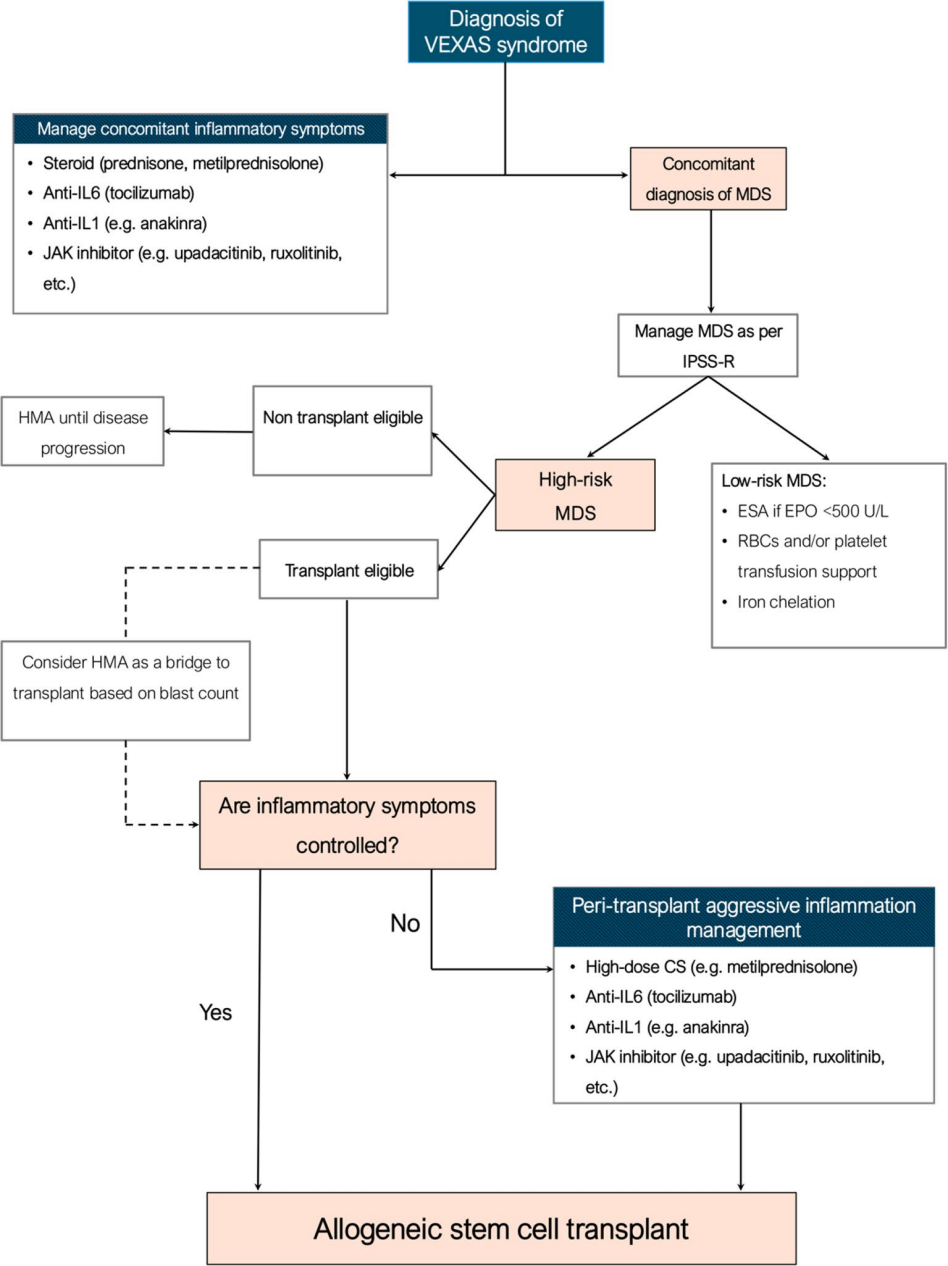
Proposed management for VEXAS syndrome. Abbreviations: CS, corticosteroids; ESA, erythropoiesis-stimulating agent; EPO, erythropoietin; HMA, hypomethylating agent; IPSS-R, revised international; RBCs, red blood cells; prognostic score system; MDS, myelodysplastic syndrome

To date, the hematologic management of VEXAS typically follows the frameworks used for associated hematologic malignancies. In practice, the IPSS-R for MDS is often used to guide therapeutic decisions; a proposed management strategy is outlined in Fig. 4 [3, 24]. Accordingly, patients 3 and 4 were treated based on their low IPSS-R risk, receiving transfusion support and ESA. In contrast, patients 1 and 2 were deemed eligible for HSCT, the only treatment capable of eradicating the *UBA1*-mutated clone. However, literature suggests that most patients, regardless of clinical severity, tend to fall into the low to intermediate IPSS-R risk categories [3, 25]. This is particularly evident in patient 2, who was referred for HSCT primarily due to uncontrolled inflammatory manifestations and persistent severe cytopenias, despite a reduced blast count. Although classified as moderate-to-high risk according to the molecular score, it is important to note that this score has not yet been validated for guiding clinical decision-making. Given the unique pathophysiological mechanisms of VEXAS compared to *UBA1*-wildtype MDS, there is an urgent need to develop dedicated prognostic models. The molecular and immunologic disruptions caused by *UBA1* mutations likely result in hematologic and inflammatory profiles that traditional scores do not fully capture. These differences may affect disease progression, cytopenias, and systemic inflammation, potentially leading to an underestimation of disease severity when using non-VEXAS models. A specific prognostic framework, incorporating VEXAS-specific markers, is critical for accurate risk stratification and tailored treatment approaches.

Current therapeutic strategies have shown limited efficacy in managing inflammatory manifestations, often requiring multiple agents to control severe inflammation [26]. In our cohort, consistent with existing literature [3, 26], all patients were treated with corticosteroids, though with suboptimal results. Patient 1 also received several DMARDs, including tocilizumab, upadacitinib, and anakinra, to achieve sufficient inflammatory control before transplant; however, intolerable toxicities required frequent discontinuation. Notably, unlike patient 2, successful transplant outcomes in patient 1 were contingent on controlling the inflammatory burden, highlighting the critical need for pre-transplant strategies (two steroid boluses were administered before stem cell infusion). In our experience, adequate pre-transplant inflammation management was crucial for transplant success (Fig. 4), as inadequate control was a key factor in the fatal outcome of patient 2. Currently, HSCT remains the only curative treatment for VEXAS, though it carries substantial risks of morbidity and mortality. However, recent data offer hope, showing a 2-year overall survival rate of 74.2% and a transplant-related mortality rate of 25.8% [11]. This suggests that HSCT could be a valuable option for selected patients, and the results of the ongoing phase II trial (NCT05027945) are eagerly awaited.

## Conclusions

Diagnosing VEXAS syndrome remains a significant challenge due to its complexity and symptom variability. Both clonal hematopoiesis and inflammation are key aspects of the disease, necessitating the management of both for successful patient outcomes. In particular, reassessing prognostic factors is crucial, as standard MDS prognostic scores may not be suitable for VEXAS. While limited by the scope of a case series, our report suggests that aggressive management of inflammation prior to transplantation is critical for success. Ultimately, an integrated and timely approach is essential to improving clinical outcomes and ensuring effective care for patients with this complex syndrome.

## Data Availability

No datasets were generated or analysed during the current study.
